# RELATIONSHIP BETWEEN CHILD MORTALITY, SOCIAL SUPPORT, AND PSYCHOLOGICAL DISTRESS AMONG WAR SURVIVORS IN VIETNAM

**DOI:** 10.1093/geroni/igae098.1769

**Published:** 2024-12-31

**Authors:** Kakada Kuy, Korinek Kim, Zachary Zimmer

**Affiliations:** Washington University in St. Louis, St. Louis, Missouri, United States; University of Utah, Salt Lake City, Utah, United States; Mount Saint Vincent University, Halifax, Nova Scotia, Canada

## Abstract

Social support has been documented as a key factor contributing to improved well-being among grieving parents in the bereavement process. However, how different forms of support interact with the experiences of child death is less understood among aging populations in post-conflict settings. This study examined the psychological impact of child death on older Vietnamese with a focus on the mediating role of social support and the moderating effect of war exposure. Data came from the 2018 Vietnam Health and Aging Study of 2,447 older adults aged 60 and older. We assessed experience of child loss by enumerating the mortality and survivorship status for all children. Mental distress was measured using the Self-Reporting Questionnaire with nine items. Social support was conceptualized along two dimensions: 1) Received social support was divided into instrumental and emotional domains; 2) perceived social network support was broken down into functional and structural aspects. Path analysis substantiated a significant relationship between child death and heightened mental distress. Emotional and labor support significantly buffered the impact of child. Moderated mediation analysis suggested that individuals with higher levels of war exposure received a noticeable benefit from emotional support. However, other forms of support did not demonstrate a significant mediating role. Results underscore the value of emotional support in this context. Findings suggest the integration of mental health support within existing social services for older adults in Vietnam with an emphasis on providing emotional support to those who have experienced child loss.

